# Proceedings from the CIHLMU International Health Symposium 2023: “One Health Approach to Neglected Tropical Diseases”

**DOI:** 10.1186/s12919-025-00317-5

**Published:** 2025-02-21

**Authors:** Kenneth Mawuta Hayibor, Doreen Ibrahim Pamba, Denise Floripes Tinga Banze, Alfred Arnold Mfinanga, Getu Ataro Hanago, Ankita Singh, Guenter Froeschl, Lisa Hoffaeller, Sarah Scholze

**Affiliations:** 1 CIH LMU Center for International Health, Ludwig-Maximilians-Universität, Munich, Germany; 2 Teaching & Training Unit, Division of Infectious Diseases and Tropical Medicine, LMU University Hospital, LMU Munich, Germany

**Keywords:** One Health, Neglected Tropical Diseases, Public Health

## Abstract

Neglected tropical diseases (NTDs) are a group of 20 conditions that affect impoverished communities and disproportionately impact women and children in tropical areas. The symposium aimed to raise awareness of NTDs and explore the One Health approach as well as actions needed to successfully combat NTDs. It featured four presentations and two panel discussions. The presentations covered topics such as the “Burden of NTDs in Low-and middle-income countries”, “Challenges in the prevention and control of NTDs: Schistosomiasis”, “One health action needed to address NTDs: the case of Neurocysticercosis”, and “The success of one health intervention in the fight against Trachoma in Kenya”. All presenters emphasized the crucial role of the One Health integrated approach in effectively and sustainably preventing NTDs.

## Background

Neglected Tropical Diseases (NTDs) encompass a diverse group of viral, bacterial, parasitic, fungal, and toxin-related conditions that persistently continue to pose a significant global health burden [1]. These diseases disproportionately affect marginalized and impoverished populations. According to a report by Lin et al., over the past two decades, the number of people affected by NTDs has increased by 72.1%, highlighting the importance of immediate attention and concerted efforts for treatment and prevention [1]. The incidence of NTDs is influenced by various factors such as geographical region, poverty, climate and season [2, 3].

Historically, significant efforts have been made to prevent, treat, and eliminate NTDs. These efforts have resulted in remarkable progress, such as the launch of the first NTDs roadmap and the London Declaration on NTDs, which have helped in the fight against these diseases [4]. Gains from these efforts include the elimination of at least one NTD from 47 countries; the development of new drug regimens; the treatment of over one billion affected individuals, and a reduction by 600 million of the people requiring intervention by 2020 compared to 2010 [5, 6]. However, climate change, natural disasters, armed conflicts and migration, as well as the unprecedented emergence of the COVID-19 pandemic in 2020 have significantly impacted efforts to tackle NTDs, disrupting ongoing interventions and diverting resources [3].

To build on past achievements and to address gaps identified in previous initiatives, the 2021–2030 NTD Roadmap and Kigali Declaration were launched [3]. The aim is to accelerate progress and synchronize efforts with the Sustainable Development Goal (SDG) 3.3, as outlined by the World Health Organization (WHO) toward ending the transmission of NTDs [7]. Recognizing the complexity and interconnectedness of NTDs, the World Health Organization has emphasized the importance of adopting an integrated and multidisciplinary strategy, known as the One Health (OH) approach, to effectively combat these diseases [3].

One Health is a holistic and integrated approach that aims to sustainably balance and optimize the health of people, animals, and ecosystems [8]. This approach is particularly relevant to NTDs, often involving the environment or animals. It provides a powerful intervention tool by comprehensively considering how human, animal, and environmental factors interact in disease processes, thus helping to combat NTDs [9]. Recognizing the importance of One Health in NTD’s control, the 2023 International Health Symposium was organized to sensitize health practitioners, researchers and the public.

## The CIH.^LMU^ International Health Symposium 2023

The CIH^LMU^ International Health Symposium is a symposia series that is running at the Center for International Health at the Ludwig-Maximilians-Universität, Munich, Germany, since 2012. It is entirely conceived and conducted by each year’s batch of candidates of the Ph.D. Program Medical Research – International Health and Master of Science Program in International Health, which have enrolled candidates from more than 40 countries so far. The symposium of 2023 focused on the "One Health Approach to Neglected Tropical Diseases" and aimed to address current issues and solutions associated with NTDs in low- and middle-income countries (LMICs). The conference brought together over 65 participants from academia, research institutes, the commercial sector, and healthcare providers, both in-person and online.

The main objectives of the symposium were to improve global understanding of NTDs, foster discussions and collaborations on prevention measures and interventions, and explore One Health strategies crucial for effectively tackling NTDs. The opening ceremony was moderated by Ankita Singh, an International Health master's student from India at LMU. She introduced the distinguished speakers, including Dr. Inge Kroidl, Dr. Bonnie Webster, Prof. Emilia Noormahomed, and Dr. Mary Asiyo-Vogel, and welcomed all participants.

Ankita gave a brief introduction to NTDs, setting the stage for the subsequent presentations and panel discussions with the invited speakers. The symposium provided a valuable platform for experts and stakeholders to share insights and exchange ideas to collaborate on effective strategies to address NTDs.

## Summary of presentations

### “Burden of NTDs in Low-and Middle-Income countries”

#### Dr. Inge Kroidl

Division of Infectious Diseases and Tropical Medicine, LMU University Hospital, LMU Munich, Germany. Trained physician and clinical researcher, specialized in internal medicine, tropical medicine and infectious diseases.

#### Overview

NTDs comprise a group of 20 conditions that primarily affect people who live in poverty, have inadequate access to sanitation, and often have close contact with domestic animals, livestock, and infectious vectors [3]. In 2023, NTDs were reported to affect 1.5 billion people worldwide, with Africa accounting for 40% of the global NTDs burden and 600 million people in need of treatment [10]. Up to 90% of the NTD disease burden in Africa is caused by about five NTDs. These are lymphatic filariasis (elephantiasis), onchocerciasis (river blindness), soil-transmitted helminthiasis (intestinal infections), schistosomiasis (bilharzia or “snail fever”), and trachoma (infectious eye disease) [3]. Controlling these diseases, which account for most infections, will be key to winning the battle against NTDs in LMICs.

The global targets and milestones for the prevention, control, elimination, or eradication of NTDs include: a 90% reduction in the number of people requiring interventions, a 75% reduction in associated disability-adjusted life years, the elimination of at least one NTD in 100 countries, and the eradication of two diseases (yaws and guinea worm) by 2030 [3].

Over the last decade, significant progress has been made in the fight against NTDs after the WHO launched its first NTDs roadmap in 2012. In 2020, 500 million fewer people needed interventions for NTDs compared to 2010, and at least one NTD has been eliminated in 40 countries, territories, and areas [3]. There have been success stories in certain countries affected by NTDs. For example, Dracunculiasis (Guinea worm disease), which used to be widespread in tropical Africa, has been eradicated in over 49 countries [11]. This disease can be prevented by treating water, using deep wells, and preventing contamination by infected persons. The last few remaining cases were reported in South Sudan, Chad, Central African Republic, and Ethiopia in 2022. Another success story is that 17 countries have successfully eliminated Lymphatic filariasis (LF), and 7 countries are currently under post-mass drug administration (MDA) surveillance as of 2020 [5]. These achievements were made possible by diagnosing the adult worm *Wuchereria bancrofti* through the detection of circulating filarial antigen using point-of-care strip tests to allow acute disease mapping and planning of governmental activities. Donation of billions of drugs by pharmaceutical companies to allow mass treatment of the whole population in endemic areas has been a pillar for the success of African governments [10].

Although some achievements have been made, overall progress has not been sufficient to eliminate NTDs as a public health problem in LMICs, especially in Sub-Saharan Africa, due to several challenges. The challenges included inadequate coverage and effectiveness of key NTD interventions, insufficient resources at all levels, and weak coordination and linkages to other sectors and health programs within countries.

### “Challenges in the prevention and control of NTDs: Schistosomiasis”

#### Dr. Bonnie Webster

Principal researcher at the Natural History Museum, London. Co-chair of the Global Schistosomiasis Alliance (GSA) diagnostics working group, a member of the GSA monitoring and evaluation working group, the GSA research group with a specific focus on One Health and also part of three WHO advisory committees namely Schistosomiasis and STH Control and Elimination, Schistosomiasis Diagnostics Technical Advisory Group and FGS Diagnostics Technical Advisory Group.

#### Overview

Schistosomiasis is an NTD that is transmitted by specific fresh-water snails, mostly affecting impoverished communities in tropical and subtropical regions of Africa, the Middle East, Asia, and Latin America [12]. The disease is commonly endemic in low-income rural areas that lack access to clean water, proper sanitation and adequate healthcare. sub-Saharan Africa accounts for up to 90% of schistosomiasis cases, resulting in an estimated 280,000 deaths annually [13].

The two main schistosomiasis species found in Sub-Saharan Africa that affect humans are *Schistosoma haematobium*, which causes urogenital schistosomiasis, and *S. mansoni*, which causes intestinal schistosomiasis. *S. intercalatum* and *S. guineensis* also cause intestinal schistosomiasis but are less prevalent [14]. Schistosomes have a complex life cycle that involves both intermediate gastropod hosts and a definitive mammalian host. Schistosoma infection progresses in three distinct disease phases, beginning with an initial dermatitis reaction following penetration of the cercariae into the skin, resulting in an allergic inflammatory maculopapular lesion. The infection may then progress to a symptomatic acute schistosomiasis stage and later chronic schistosomiasis [15].

Schistosomiasis control programs in Africa mainly focus on community-based preventive chemotherapy [15]. Most NTD control programs use praziquantel for effective morbidity reduction through mass drug administration. Each year, endemic countries in Africa are provided with donated praziquantel to treat millions of school-aged children [16]. However, compliance with treatment is a significant challenge, particularly among individuals residing in low socioeconomic areas. This lack of adherence may be due to the fear of adverse effects, the absence of disease symptoms, or even the stigmatization of symptoms [17, 18], which are often viewed as a normal sign of puberty and not requiring treatment [19].

There has been a significant increase in efforts to eliminate schistosomiasis in the past decade, with the World Health Organization (WHO) setting the goal of transmission interruption in endemic African countries by 2030 [3]. Governments of various countries have prioritized the control of NTDs by implementing measures such as MDA, snail control, improving sanitation, and providing access to safe, clean water. However, in Sub-Saharan Africa, preventive chemotherapy remains the primary intervention [15].

## Panel Discussion 1 on burden and challenges in the prevention and control of NTDs in LMIC

### Moderator: *Kenneth Hayibor, Ghana,* PhD student, CIH^LMU^

#### Panelists: Dr. Inge Kroidl and Dr. Bonnie Webster

- Key strategic interventions to accelerate the prevention, control, elimination, and eradication of NTDs in LMICs include health system strengthening, mass drug distribution, continuous education, and community engagement. A number of these strategies have been implemented in most endemic countries, especially in sub-Saharan Africa and most have been proven to be effective [9]. These strategies are based on the WHO recommendation to combat NTDs as a group of diseases based on a combination of five public health interventions: (i) innovative and intensified disease management; (ii) preventive chemotherapy; (iii) integrated vector management; (iv) veterinary public health; and (v) access to water, sanitation, and hygiene [20].
- With the increase in migration and international travel, NTDs are no longer confined to specific geographical areas. They are now increasingly being observed outside tropical regions. In the Western world, NTDs are primarily diagnosed among travelers coming from or returning to endemic countries. It is therefore not only important to understand the modes of transmission within endemic regions, but also outside. Moreover, eradication programs targeting NTDs in endemic areas may reduce the disease prevalence among migrants and travelers in non-tropical Western countries [21].
- Individual and social limiting factors such as poor education and sociocultural beliefs are very important drivers of NTDs. Entrenched cultural norms and practices influence human and community perceptions of the disease-environment interaction and also determine social behavior and response thereby preventing the control of NTDs. In most NTDs endemic countries, it is not uncommon to find NTD infections being attributed to “charms or witchcraft”. In Benin, infection with Buruli ulcer is attributed to trespassing on another person's property [22]; while in Northern Ghana, infection with lymphatic filariasis is attributed to witchcraft [23]. These attributions are mainly due to a lack of knowledge and poor education, which leads to stigmatization. Adequate education of the population at risk is an important contribution to the fight of controlling NTDs.
- Vector control by use of hazardous chemicals to prevent NTDs should be approached with caution. For instance, in the quest to control schistosomiasis in Malawi, chemicals used to kill snails affected the fish population in the water [24]. Biological control methods are better, for example the use of large fish that eat snails or compete with them for food. However, the WHO recommends that when using chemical-based molluscicide, field use of molluscicides in schistosomiasis control programs' operative manual should be followed [25].
- Climate change and the burden of NTDs are closely linked. The effect of climate change on the burden of NTDs can be bidirectional: drought may decrease hosts such as snails, and flood increases the transmission of NTDs. Climate change may influence the emergence and re-emergence of multiple NTDs, particularly those that require a vector or intermediate host for transmission [26].
- The COVID-19 pandemic has severely impacted the control of NTDs. In response to the COVID-19 pandemic, the WHO recommended in 2020, that NTD control programs postpone all activities related to active case detection, community-based surveys, and mass drug administration [27]. The COVID-19-induced interruption in terms of delay in achieving the elimination of seven NTDs might in some cases extend beyond the duration of the interruption [28].

### “One health actions needed to address NTDs: the case of neurocysticercosis”

#### Professor Emilia Noormahomed

Professor of Human Parasitology at the University of Eduardo Mondlane in Maputo, Mozambique. She is also an invited Professor at the University of California San Diego in the United States of America and a Senior Researcher at the Mozambique Institute for Health Education and Research (MIHER) in Mozambique. Her research primarily focuses on defining clinical and epidemiological aspects of *T. solium* cysticercosis and neurocysticercosis and other poverty-related diseases, on the development of point-of-care diagnostic tools for infectious diseases including the study of bidirectional actions of these pathogens with HIV.

#### Summary of Presentation

Neurocysticercosis (NCC) is the most frequent helminthic infection of the central nervous system and a leading cause of acquired epilepsy worldwide [29]. It is currently a growing public health problem in the United States. As millions of people from Latin America have immigrated to the United States in recent years, neurocysticercosis has become an increasingly important cause of seizures in the United States [30]. It is, however, endemic in most developing countries where conditions favoring the transmission of this disease, including swine breeding under poor sanitary conditions, poverty, and illiteracy, are met [31]. The disease occurs when humans accidentally become hosts of *Taenia solium* by ingesting its eggs from contaminated food or, most often, directly from a *Taenia* carrier by the fecal-to-oral route. Seizures are the most common clinical manifestation, but many patients also present focal deficits, intracranial hypertension, or cognitive decline. Lesions are predominantly intracranial, although other predilection sites like ophthalmic localizations and muscular calcifications exist [32].

A single therapeutic approach is not expected to be useful in every patient with neurocysticercosis [33]**.** The use of cysticidal drugs (albendazole and praziquantel) has been shown to reduce the parasite load within the central nervous system and to improve the clinical prognosis of most patients with neurocysticercosis [34]. Eradication of the disease can be achieved through the implementation of control programs against all interrelated steps in the life cycle of *T. solium* and this can be done through adopting the One Health approach.

A One Health approach can help control NCC by addressing human and animal health, environment, community livelihood, capacity development, and policy development. The actions needed include recognizing infected meat, avoiding open defecation and free-roaming pigs, implementing preventive measures, adopting safe cooking practices and proper pig husbandry practices, increasing knowledge and awareness about the diseases, training community health workers and health professionals to recognize, diagnose, and treat affected people, and developing policies to treat epilepsy and share knowledge and best practices.

These actions were used in Mocuba, located in Zambézia province, which has one of the largest pig populations and a high prevalence of NCC in Mozambique. After implementing these actions, most community members had an increased knowledge of recognizing infected meat, ways to prevent it, and the need to avoid stigma and discrimination against epileptics. Over 300 different health professionals were trained in the treatment of epilepsy, and drugs for the treatment were made available in all health units. More than 7,000 people benefited from the treatment of epilepsy.

### “The success of one health interventions in the fight against trachoma in Kenya”

#### Dr. Mary Nyamasi Asiyo-Vogel

An eye doctor who co-founded the "Augenpraxisklinik Lübeck" and also provides eye care services in rural Western Kenya. She is a renowned lecturer on tropical ophthalmology and provides lectures on the subject in courses on tropical medicine held at the Bernhard Notch Institute for Tropical Medicine in Hamburg and the Ludwig-Maximilians-Universität in Munich.

#### Summary of presentation

Trachoma is the world’s leading infectious cause of blindness and one of 20 neglected tropical diseases (NTD). It accounts for 1.4% of blindness worldwide. About 7 million people live in 12 central Kenyan counties where trachoma is endemic due to dry climate and the pastoral and nomadic lifestyle [35]. About 53,000 people in Kenya are currently infected with the disease. Subsequent baseline surveys conducted in 2007–2012 showed that, of the 47 counties in Kenya, 12 counties with a combined population of approximately 7 million were confirmed to be trachoma endemic. In 2013, the Ministry of Health estimated that 41,500 individuals in these 12 counties had trachoma at stage trachomatous trichiasis and were at risk of progressive blindness, requiring surgery to prevent it resulting in the initiation of the Kenya Trachoma Elimination Programme (KTEP) in 2014, a 5-year program to eliminate trachoma as a public health problem by 2019, using the Surgery, Antibiotics and Facial hygiene (SAFE) strategy [36]. The goal to eliminate Trachoma as a Public health problem in Kenya by 2020, as per the World Health Organization’s target has now been postponed to 2023 to clear the remaining trachoma trichiasis backlog, which stands at around 5,000 in 12 counties [37].

To reach the 2020 targets for the global trachoma elimination by 2025, it will be necessary to scale up the SAFE strategy to full implementation in all endemic districts. Implementation of all components of the SAFE strategy relies on multiple partnerships in various sectors; especially on an adequate number of trained eye health surgeons equipped with appropriate surgical tools, water, sanitation, and education. Ownership, funding of all elements, and sustainability are essential to achieving the elimination target. The way forward for Kenya in the trachoma elimination program is to clear the remaining trachoma trichiasis backlog, which stands at around 5,000 at the moment. That is 5,000 people who require urgent surgeries to avert blindness in the 12 trachoma-endemic counties.

## Panel discussion 2 on One Health actions and interventions for the prevention and control of NTDs in LMICs

### Moderator: *Denise Banze, Mozambique, *PhD student, CIH^LMU^

#### Panelists: Dr. Mary Asiyo-Vogel and Prof. Emilia Noormahomed

- Countries like Nigeria have demonstrated the importance of local community and government ownership of NTD control programs for sustainable outcomes [38, 39]. This approach ensures long-term commitment and resources dedicated to trachoma control efforts, rather than relying solely on external donations.
- When it comes to tangible interventions such as building toilets, it is recognized that they may not be effective on their own. Dr. Asiyo-Vogel provided examples from Tanzania, Rwanda, and Kenya, emphasizing the significance of understanding and integrating local cultures into strategies. For instance, in certain communities like the Maasai people, latrines may not be used if women and children are present [35]. Hence, cultural sensitivity and engagement are crucial for designing effective interventions. It is preferable to share responsibility with the community and allow them to contribute feasible solutions based on their cultural beliefs.
- Regarding One Health interventions targeting both humans and animals, there is a question about the cost-effectiveness of animal-focused programs, particularly for controlling NCC. These programs have been found to be cost-effective. However, creating awareness about the disease among the community and educating them to avoid consuming raw pig meat, a common risk factor for NCC transmission, should be the initial step [40]. This highlights the importance of an integrated approach that considers all three aspects of One Health: humans, animals, and the environment.
- Misdiagnosis of NCC with other conditions such as pre-eclampsia and epilepsy is common due to a lack of awareness in the community and among primary healthcare providers [41]. To address this, it is recommended to gather evidence about NCC, present it to the community and policymakers. Integrating NCC awareness with existing programs like mental health initiatives or intestinal parasite control programs can be effective since prevention and treatment strategies for these diseases often overlap.
- Establishing multidisciplinary networks for the One Health approach to NTD control is becoming increasingly important. It requires collaboration with dedicated individuals and involvement of policymakers. Many health systems still rely on structures established during the colonial era, which may not adequately address NTDs. Therefore, revising policies, developing robust health systems tailored to local challenges, and integrating NTD control efforts into those systems are essential steps to enhance the effectiveness of interventions.

## Conclusion

To effectively combat NTDs, a comprehensive and multidisciplinary One Health approach is essential. This approach recognizes the critical interfaces between human, animal, and environmental health. As demonstrated by the various speakers, the One Health approach has proven successful in multiple settings. Implementing this approach should include diverse strategies such as prevention, effective treatment, and addressing healthcare disparities. Furthermore, the active involvement of local communities is vital in overcoming cultural barriers and societal views that impede the control of NTDs.

## Data Availability

Symposium speakers’ presentations are available upon request.
